# BioBanking as the central tool for translational medicine *CTM issue 2013*

**DOI:** 10.1186/2001-1326-2-4

**Published:** 2013-02-06

**Authors:** György Marko-Varga

**Affiliations:** 1 Clinical Protein Science & Imaging, Biomedical Center, Department of Measurement Technology and Industrial Electrical Engineering, Lund University, BMC C13, Lund, 221 84, Sweden; 2 First Department of Surgery, Tokyo Medical University, 6-7-1 Nishishinjiku Shinjiku-ku, Tokyo, 160-0023, Japan

## Abstract

**The impact of mapping the human proteome:**

Globally, the health care organizations are under resource and cost constrains due to the increasing number of patients that are due to a fast increase of the 65+ age group requiring extensive medical hospitalization and treatments. Hospitals worldwide strive to seek the best cure for patients, suffering from various diseases. A consequence of these global changes, of healthy populations in relation to patients forms the basis for the build of large and centralized biobank facilities, with strategies where the search for an understanding of diseases at a molecular level is at heart. The efforts made lies within large governmental resource allocations where patient centers are collecting samples from clinical study participants in order to try to discover universal expression patterns and molecular signatures of disease and disease stages. Most developments in this area are aimed towards the discovery, and understanding diagnosis implementations. By providing the right treatment alternatives for patients care, at the right time point *i*.*e*., at a given disease stage development becomes a major goal where pharmaceutical industry, academia and the health care sector joins forces in large clinical epidemiological, population-, and disease based studies. This becomes a clear strategic link to the enhancement and prospects for personalized medicines and target directed diagnosis developments (Companion Diagnostics), which require coordinated efforts across a wide range of disciplines. Currently, companion diagnostics is at the core of the personalized medicine paradigm shift. It will identify patients who are most likely to benefit from a particular therapeutic product, as well as identify patients likely to be at increased risk for serious adverse reactions as a result of treatment with a particular therapeutic agent. It is predicted that more than half of all new drugs will require a companion diagnostic, which opens up for an endeavor for Proteomics research implementations.

## Biobanking and bio-repositories

Global healthcare changes forms the basis for the rapid development of centralized large scale biobank facilities, with strategies to improve on patient status at hospitals where limited resources are at hand [1,2].

Finding the [atient group of responders to a given medical treatment is hospitals today is a major challenge. Especially as we are entering a period of targeted drug treatments. The consequence is that large clinical epidemiological, population-, and disease based studies are undertaken to get an overall higher efficacy as well as an improved safety assessment where Companion Diagnostics will play a major role [3-5].

Biobanks were found to be within the ten ideas changing the world right now [6]. Biobank repositories requires biobank sustainability where adequate staffing, including pathology support is integrated into the health care organization of hospitals.

**Figure 1:**
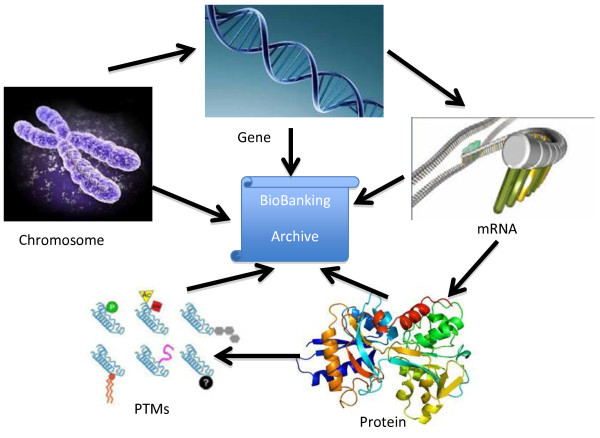
Schematic illustration of the molecular targets and drivers that will form the basis for future drugs and diagnostics in hospitals throughout the world.

The molecular value of information that is the treasure of a biobank is illustrated in Figure 1.

**Figure 2:**
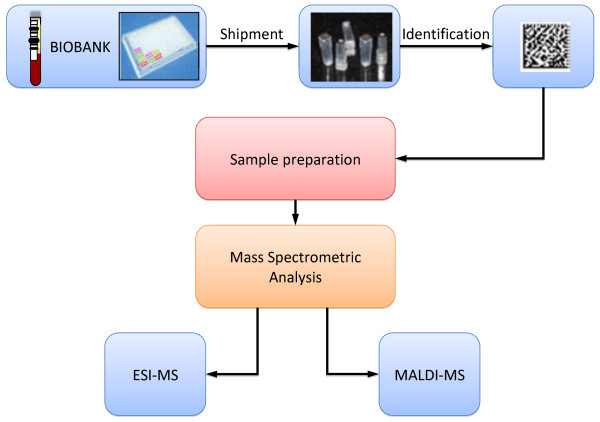
Biobank work flow where large scale Biobanks are utilized for high-throughput protein sequencing using mass spectrometry platforms.

Blood fractions from patients comprising whole blood, serum, plasma (EDTA, citrate, and heparin) are being stored and processed by automated processing in -80C storage units. High-density sample tube systems are applied to maximize the sample output per patient utilizing 384-rack systems [7]. Bar code assignments ensure the uniqueness of each and every sample that is used within the analysis process generating both MS-MS and antibody read-outs. The workflow and processing of Biobank samples to generate extensive protein sequence information in health and disease is outlined in Figure 2.

The current status of healthcare requests and demands can be summarized by that currently more than 7,000 rare diseases affect human mankind and less than 200 have available pharmacotherapy [8].

Considering an estimated number of 20,300 proteins within the Human Proteome, coded by the genome, will increase and reach into several million proteins after cellular processing, the key protein targets to be considered is a real challenge. Thus, the C-HPP consortia deliveries over the next coming years will be instrumental in these development programs.

These difficult healthcare issues will entail large number of biobank samples with ample statistical power. Worldwide biobank archives will need to work together and exploit these cohort sample assets in order to generate value in future science milestones that will transform value to the patient.

Biobanks of human patient samples tissues and blood fractions are increasingly recognized as major assets in disease research [9,10]. These new sample archives are built within both academic, commercial health care, the drug development industry, as well as within the government regulating agencies.

Biobank samples are increasingly used as a major resource for scientists to access unique patient samples for medical research and patient treatments. Many studies within clinical proteomics utilize stored samples contained in biobanks to measure specific protein expressions. For Biobank samples stored in either −20°C or −80°C (the preferable temperature), there is still today a debate on how to reach a consensus on how best to provide:

• Sampling procedures and protocols

• Management and storage conditions

• Specific sample and Data assignments to sample tubes linked to the barcode identifier

• LIMS and IT/IS solutions allowing sample tracking ability

All these efforts are performed in order to reach goals where these efforts are turned into value for the patient. Standardizations of quality control of samples being processed for storage as well as retrieval of stored samples are important goals in order to support the development of both drug targets as well as diagnostic tools such as biomarkers.

Currently the highest unmet need in modern healthcare is the monitoring and prediction of response to treatment. The optimization of drug treatment that easily can be adjusted for by scheduling the dosing, discontinuing, the drug treatment, and thereby optimize and improve on the safety and/or efficacy of a targeted drug treatment [11,12]. Consequently, the status today is that the lack of available high quality sample collections with a wide range of disease cohorts currently is a rate-limiting step for drug development, medical research and novel diagnostics.

Ultimately, the C-HPP global project initiative will aid by increasing the awareness within the proteomics community regarding the basic concepts of clinical samples. Understanding and standardizing the qualitative requirements of clinical samples in order to generate protein analysis data that can be globally shared and used for future developments within our society will also form the basis for improving on the data quality of proteomic studies. Currently, the attritions of drugs are 51% due to efficacy, and 20% for safety, in relation to as low as 1% due to pharmacokinetic properties [11]. This means that the drug development processes that include drug design and synthesis followed by physicochemical property optimization of drugs, is currently the strong part of pharma pipeline strategy. Consequently, it is imperative to understand the role of drugs and what they do in patients. Areas such as:

• The disease link

• The link of Mode of drug action to the patho-physiology

• Disease mechanisms that relates to molecular pathways

• Genetics and selected Protein targets with high drug affinities

That needs to be aligned with the:

• Patient Stratification selecting the right phenotype (s)

• Potential responders within the patient cohort

• Individual risk assessments

The targeted Personalized Medicine developments ultimately will need to align with the increasing safety demands that are key for preventive medicine and early treatment. Another challenge for new drug developments, where the C-HPP deliveries can play a role are disease phases and complex patho-physiology that needs to be understood for any given treatment.

## Conclusions

Translating basic science to discover new and better treatments is an enormous challenge where the understanding of protein function(s), both as targets as well as biomarkers will bring great value in the near future to come. This includes the missing proteins (currently about 30% of the human proteome) that have not yet been identified on a protein level but predicted from DNA, or identified as transcripts. Another area where the C-HPP will have an important role to play is within the area of the future healthcare with drug and diagnostic developments for novel treatments of complex diseases. The future direction envisions that large studies will be accompanied by, biological-, and molecular- information and corresponding material from large numbers of patients and healthy persons, where the protein data from large biobank archives will play a mandatory role. Accordingly, there is a call for further develop value and treasure that biobanking holds with a global approval on the quality, standardization and the organization of modern units being developed for future use. There are also demands and expectations from national agencies on quality for a successful usage of biobank materials, particularly in the area of novel drug developed with accompanying diagnostics introduced into the hospitals.

## References

[B1] Marko-Varga G Understanding drug uptake and binding within targeted disease micro-environments in patients: a New tool for translational medicine Clin Translat Med 2012 1 8 10.1186/2001-1326-1-8 PMC3560985 23369501

[B2] LaBaer J Improving international research with clinical specimens: 5 achievable objectives J Proteome Res 2012 11 5592 22998582 10.1021/pr300796m PMC3640360

[B3] Hamburg MA Collins FS The path to personalized medicine N Engl J Med 2010 363 301 10.1056/NEJMp1006304 20551152

[B4] Marko-Varga GA Végvári Á Fehniger TE Public service review European Union 2011 21 250 252

[B5] Hewitt RE Biobanking: the foundation of personalized medicine Curr Opin Oncol 2011 23 112 10.1097/CCO.0b013e32834161b8 21076300

[B6] Park A Time 2009

[B7] Malm J Large scale biobanking of blood - the importance of high density processing procedures J Proteomics 2012 76 116 22580359 10.1016/j.jprot.2012.05.003

[B8] Bevilacqua G The role of the pathologist in tissue banking: European consensus expert group report Virchows Arch 2010 456 449 10.1007/s00428-010-0887-7 20157825 PMC2852521

[B9] Paik YK The chromosome-centric human proteome project for cataloging proteins encoded in the genome Nat Biotechnol 2012 30 221 10.1038/nbt.2152 22398612

[B10] Miller G Is pharma running Out of brainy ideas? Science 2010 329 502 10.1126/science.329.5991.502 20671165

[B11] Wagner JA Williams SA Webster CJ Biomarkers and surrogate End points for Fit-for-purpose development and regulatory evaluation of New drugs Clin Pharmacol Ther 2007 81 104 10.1038/sj.clpt.6100017 17186007

[B12] Végvári Á Welinder C Lindberg H Fehniger TE Marko-Varga G Biobank resources for future patient care: developments, principles and concepts J Clin Bioinformatics 2011 1 24 10.1186/2043-9113-1-24 PMC3197484 21923917

